# In Situ Determination of Droplet and Nanoparticle Size Distributions in Spray Flame Synthesis by Wide-Angle Light Scattering (WALS)

**DOI:** 10.3390/ma14216698

**Published:** 2021-11-07

**Authors:** Simon Aßmann, Bettina Münsterjohann, Franz J. T. Huber, Stefan Will

**Affiliations:** 1 Lehrstuhl für Technische Thermodynamik (LTT), Friedrich-Alexander-Universität Erlangen-Nürnberg (FAU), Am Weichselgarten 8, 91058 Erlangen, Germany; simon.assmann@fau.de (S.A.); bettina.muensterjohann@fau.de (B.M.); franz.huber@fau.de (F.J.T.H.); 2 Erlangen Graduate School in Advanced Optical Technologies (SAOT), Friedrich-Alexander-Universität Erlangen-Nürnberg (FAU), 91052 Erlangen, Germany; 3 Cluster of Excellence Engineering of Advanced Materials (EAM), Friedrich-Alexander-Universität Erlangen-Nürnberg (FAU), 91052 Erlangen, Germany

**Keywords:** spray flame synthesis, flame spray pyrolysis, droplet size distribution, particle size distribution, TiO_2_-nanoparticles, spherical particles, fractal aggregates, elastic light scattering, wide-angle light scattering, multivariate data analysis

## Abstract

The investigation of droplet and nanoparticle formation in spray flame synthesis requires sophisticated measurement techniques, as often both are present simultaneously. Here, wide-angle light scattering (WALS) was applied to determine droplet and nanoparticle size distributions in spray flames from a standardized liquid-fed burner setup. Solvents of pure ethanol and a mixture of ethanol and titanium isopropoxide, incepting nanoparticle synthesis, were investigated. A novel method for the evaluation of scattering data from droplets between 2 µm and 50 µm was successfully implemented. Applying this, we could reveal the development of a bimodal droplet size distribution for the solvent/precursor system, probably induced by droplet micro-explosions. To determine nanoparticle size distributions, an appropriate filter and the averaging of single-shot data were applied to ensure scattering from a significant amount of nanoparticles homogeneously distributed in the measurement volume. From the multivariate analysis of the scattering data, the presence of spherical particles and fractal aggregates was derived, which was confirmed by analysis of transmission electron microscopy images. Monte Carlo simulations allowed determining the distribution parameters for both morphological fractions in three heights above the burner. The results showed relatively wide size distributions, especially for the spherical fraction, and indicated an ongoing sintering, from fractal to spherical particles.

## 1. Introduction

During recent decades, gas phase processes have developed into a commonly used technique for the synthesis of functionalized nanoparticles from a wide variety of materials and compounds [1,2,3,4,5]. Spray flame synthesis (SFS), in particular, offers significant advantages compared to other gas phase techniques, as a large variety of common and affordable substances can be utilized in a continuous production process, which can be easily scaled up for industrial application. Thus, the economical production of high-purity product particles in a wide range of different materials and morphologies, and with defined functionality, is feasible [1,3]. In this process, non-volatile precursor materials are dissolved in a suitable solvent and atomized in a flame, initiating droplet evaporation, thermal decomposition of the precursor, and chemical formation of the desired particle material. The formation of particles is strongly dependent on the chemical and physical properties of the materials used (e.g., their chemical composition, boiling and decomposition temperatures, and specific enthalpy) and the conditions of the spray flame (e.g., the atomization quality of the solvent and the temperature field of the flame), and this can lead to various particle morphologies, ranging from fractal-like aggregates to spherical particles [3,6]. If flammable solvents are used, the additional energy contribution from their combustion results in higher temperatures and, thus, accelerated evaporation and reaction processes. The process is then usually referred to as flame spray pyrolysis (FSP) [1,3,4]. A comprehensive investigation of the underlying processes of FSP is crucial for successful industrial scale-up, and this is systematically carried out within the framework of the priority program on ‘Nanoparticle Synthesis in Spray Flames: Spray-Syn: Measurement, Simulation, Processes’ (SPP1980) of the German Research Foundation (DFG), using a well-defined and standardized FSP burner setup, the SpraySyn burner [2,7,8].

For a comprehensive investigation of nanoparticle characteristics from FSP processes, a variety of measurement methods are suitable. Invasive methods, such as scanning mobility particle sizing (SMPS), X-ray diffraction (XRD), transmission electron microscopy (TEM), and particle mass spectrometry (PMS) provide substantial and comprehensive data, but require sampling of the particles within the process and, thus, may influence the process and the derived quantities themselves [9,10,11]. Moreover, the highly turbulent character of the process might lead to biased results. In situ methods can overcome this drawback. As an example, small/wide-angle X-ray scattering (SAXS/WAXS) allows determining the morphology and size of particles in a flame; however, very brilliant X-ray sources like synchrotrons are required to resolve these characteristics with acceptable temporal resolution [12]. Laser-based in situ methods such as laser-induced incandescence (LII), Raman spectroscopy, and elastic light scattering (ELS) can overcome this drawback [3]. While Raman spectroscopy allows, e.g., determining the crystalline structure of the particles synthesized, it is not capable of inferring their size and morphology [13]. With time-resolved LII the primary particle size is accessible, yet the technique is limited to materials with high evaporation temperature and suitable optical properties [14].

ELS is typically based on the detection of the angular distribution of scattered light, which is characteristic of the size and structure of the scattering particles [15,16]. Imaging approaches using multiple cameras with different detection angles have recently been improved for single-shot acquisition and aggregate sizing [17]; however, their application to a real turbulent FSP-process has not been demonstrated so far. Moreover, these approaches suffer from a limited angular resolution, which causes problems in the case of droplet scattering (hard to distinguish from particle scattering in the case of limited data density) or in the determination of particle size distributions. Within recent years, the wide-angle light scattering (WALS) approach has been developed and applied to the investigation of nanoparticle characteristics in flames and aerosols, obtaining size distributions of spherical particles and fractal aggregates [18,19,20,21,22]. More recently, the WALS technique was successfully applied to the determination of droplet size distributions in the SpraySyn burner, making it a promising technique for the comprehensive investigation of both droplet and nanoparticle formation in FSP [23]. In our previous work, however, spray flames of pure solvents, inhibiting nanoparticle formation, were used to demonstrate the applicability of the technique for the droplet formation of the FSP process, without the interference of nanoparticle scattering [23].

A precursor-laden solvent induces the desired particle formation, and eventually regions along the height above burner surface (HAB), containing both nanoparticles and droplets, are formed. Deriving size parameters for both fractions from scattering data with WALS requires proper data filtering and optimized evaluation algorithms, which are presented in this work. To that end, we improved the evaluation routine for droplet sizing in spray flames presented in [23], making it applicable to scattering from spherical droplets, even with the presence of nanoparticles in the WALS measurement volume. Here, similar to our previous work, a short-pulsed laser source is required to capture the scattering patterns of single droplets in the micrometer range. An appropriate derivation of nanoparticle characteristics from WALS data requires a sufficiently homogeneous distribution of particles within the measurement volume at the time of measurement, which is challenging when using a pulsed laser source in combination with a turbulent process such as the FSP. Therefore, we implemented appropriate data filtering and averaging of the scattering data stemming from the nanoparticles prior to the evaluation. Sophisticated algorithms were applied to the data obtained, to derive the particle size distribution parameters by inverse analysis. Measurements were carried out at the HAB between 20 mm and 120 mm in spray flames of pure ethanol (EtOH) and of EtOH containing titanium isopropoxide (TTIP) with a molarity of 0.1 M, for the production of titanium dioxide (TiO_2_) nanoparticles. Thereby, the entire formation process of nanoparticles in spray flames can be investigated with one measurement method. Besides the measurement of droplet sizes, this approach permits the distinction between spherical particles and fractal aggregates, which is crucial for a better understanding of the underlying particle formation pathways and, eventually, the application of SFS for nanoparticles with defined functionality.

## 2. Materials and Methods

### 2.1. Experimental Setup

In this work, we used the same SpraySyn-burner (University of Duisburg-Essen, Duisburg, Germany) and optical measurement setup as described in our previous work. In the following, only a brief summary is given, for a detailed description we refer the reader to references [7,23]. The key element of the burner is a concentric two-fluid nozzle system, consisting of two parts: the capillary for the precursor solution, which is contained in the inner ring of the nozzle, and an annular slit, through which a dispersion gas initiates the atomization of the precursor solution, which is contained between the capillary and the outer ring of the nozzle. The nozzle is surrounded by a bronze sinter-matrix: premixed gases for the lean pilot flame flow through its inner region, while nitrogen (N_2_) or air flows through its outer region to shield the flame from ambient gas. The gas flow rates are controlled by mass flow controllers: for the dispersion gas, we used a flow rate of 10.0 slm (standard liters per minute) of oxygen (O_2_, purity 2.5), for the pilot flame a mixture of 2.0 slm methane (CH_4_, purity 2.5) and 16 slm O_2_ (purity 2.5), and for the sheath gas pressurized and dried air at 120 slm. In previous work, it could be shown that using air instead of N_2_ as a sheath gas does not significantly influence the droplet formation and evaporation behavior [23]. We assumed a similar negligible influence of the sheath gas on the formation of nanoparticles. TiO_2_-nanoparticles were generated using a 0.1 M solution of TTIP (purity ≥ 98%, Merck KgaA) dissolved in pure EtOH (absolute for analysis EMSURE^®^, Merck KgaA, Darmstadt, Germany). The precursor solution was continuously pumped at 2.0 mL/min by a twin syringe pump. The correct operation of the burner was ensured by applying the benchmark procedure described by Schneider et al. prior to the measurements [7].

For the measurement of droplet and particle size distributions, wide-angle light scattering (WALS) was used [18,19,20,21,22,23]. As a light source, a pulsed Nd:YAG laser (Q-Smart 450, Quantel Technologies, Les Ulis, France) was employed at a wavelength of 532 nm, with a maximum pulse energy of 190 mJ, at a repetition rate of 10 Hz, and a pulse width of ~6 ns. First, the laser beam is directed through both a half-wave plate and a thin-film polarizer to adjust the pulse-energy in the measurement volume. The laser is then shone through a circular aperture of 1 mm diameter, to obtain a near top-hat laser profile, ensuring homogeneous illumination in the measurement volume. To minimize beam blurring due to diffraction, the aperture is imaged into the measurement volume using a 4*f*-setup, consisting of two lenses with *f* = 300 mm. Before entering the measurement volume, the polarization in the measurement volume is set to vertical using a second half-wave plate and the beam polarization is purified using a Glan laser polarizer (Thorlabs GmbH, Bergkirchen, Germany). The ellipsoidal mirror has a focal length of Δ*f* = 600 mm and the laser beam shines through the mirror’s first focal point. The surface of the ellipsoidal mirror is imaged on a CCD-camera (Pike F-100B, Allied Vision Technologies GmbH, Stadtroda, Germany) with an aperture and a lens (*f* = 12.5 mm) in the second focal point of the mirror. The combination of the laser diameter and the size of the camera aperture define the spatial extension of the measurement volume. In this work, the laser diameter was set to 1 mm and the camera aperture to *f*/2.0, to keep the measurement volume constant for all examinations. Ambient light and flame luminosity are blocked by a 535 nm bandpass filter (OD 4, bandwidth 10 nm, Edmund Optics GmbH, Mainz, Germany) in front of the camera lens. 

The obtained ring-shaped camera image is then divided into angular sectors, between a 10° and 170° scattering angle, following the procedure of Huber et al. [20], with an angular resolution of 0.2° for the scattering of droplets and 1.0° for the scattering of particles. All pixels within a sector are averaged, resulting in a corresponding angular distribution of the scattering intensity. Due to the averaging process, high signal-to-noise-ratios (SNR) are obtained, even from single-shot scattering data. To account for the angular-dependent size of the measurement volume relevant for the evaluation of nanoparticle scattering, a calibration was carried out using sulfur hexafluoride (SF_6_) instead of N_2_, due its larger scattering cross section [19]. Measurements were performed at different HAB, between 20 mm and 120 mm, along the center axis of the burner. An investigation of the lower regions of the spray flame was not feasible, as atomization of the liquid was still ongoing and, thus, a large number of droplets, along with many non-spherical structures, were present, resulting in unevaluable scattering data [24]. For each measurement position, between 2000 and 6000 single-shot images were acquired. To verify results from the particle scattering, thermophoretic sampling in combination with TEM image analysis was performed on particle samples of the EtOH/TTIP-flame at a 120 mm HAB [25,26].

### 2.2. Evaluation of Scattering Data from Microdroplets

**Figure 1:**
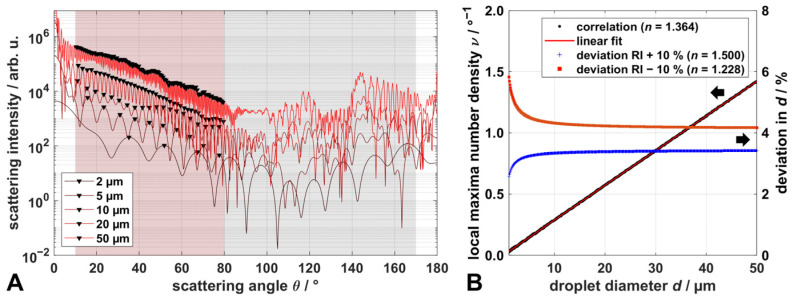
(**A**): Scattering data of spherical EtOH droplets calculated with Mie theory for diameters *d* between 2 µm and 50 µm for vertically polarized light with a wavelength of 532 nm (the periodically occurring local maxima are marked with ▼ for each data set, the region accessible with WALS is shaded, the evaluation region of WALS is additionally highlighted in red); (**B**): linear correlation curve between droplet diameter *d* and local maxima number density *ν* (see text) derived from calculated scattering data and deviation, i.e., over- or underestimation, respectively, of the droplet size for a refractive index variation of ±10% based on the refractive index of EtOH.

The resulting scattering data were further processed to determine droplet and particle size distributions. In comparison to our previous work, here the precursor TTIP was added to the solution, leading to the formation of TiO_2_-nanoparticles. Thus, superimposed scattering data from both droplets and nanoparticles might occur. To evaluate these data, we improved the evaluation routine for deriving droplet size distributions. Scattering data of spherical droplets in the lower micrometer range exhibit an oscillating signal, with periodically occurring local maxima and minima with increasing frequency for larger droplet sizes, especially in the forward scattering regime between 10° and 80° (Figure 1A).

**Figure 2:**
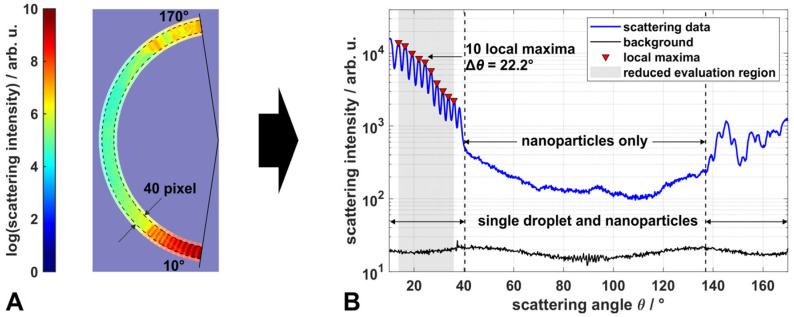
Example determination of the droplet size from superimposed scattering data of TiO_2_-nanoparticles and an EtOH/TTIP droplet: From the scattering image of a mirror section ((**A**); data in logarithmic scaling and displayed in false color), scattering data (blue) are generated and local maxima (red) in the evaluation region are determined ((**B**); regions with scattering of droplets and/or particles are labeled correspondingly, background level is shown in black). The droplet size is then determined from the linear correlation curve depicted in Figure 1B.

Mie scattering signals were calculated for vertical polarized light at 532 nm wavelength using the same MATLAB based code for spheres of diameter *d*, based on Bohren and Huffmann and refractive indices for EtOH (*n* = 1.364) and ambient atmosphere (*n* = 1.000), as in our previous work [23,27]. With the MATLAB embedded parameterized *peakfinder* function, the angular positions of local maxima can be determined very precisely. With the parameter ‘minimum peak prominence’ (MPP), a minimum threshold can be set, so that only peaks above this threshold are registered as local maxima. Hence, a suitable choice of this parameter is crucial, especially for the measurement data to minimize errors in the evaluation, such as detecting noise as local maxima (overestimation of *d*) or an incomplete detection of local maxima within the angular evaluation region (underestimation of *d*). For simulated scattering data, the MPP was set to 0.01 and the algorithm was used on the logarithm of the scattering intensity, as it showed a more robust behavior than on the absolute scattering intensity. In the angular region of interest, between 10° and 80° (evaluation region, highlighted red in Figure 1A), all local maxima for sphere diameters between 2 µm and 50 µm were determined very accurately for the simulated scattering data, obtaining the linear correlation $d=[35.26^{{}^{\circ}}\cdot \nu -0.136]$ µm between *d* and the number density of local maxima $\nu =(N_{\max}-1)/\Delta \theta$, with the number of local maxima $N_{\max}$ per angular range Δθ (Figure 1B). This correlation can be used to derive droplet sizes from measurement data, by detecting the local maxima in the scattering patterns. As calculated data show very periodically occurring local maxima for an individual droplet size, the number density of local maxima also remains constant for a reduced angular evaluation region and, thus, the droplet sizes of measured data can be derived, even if only two distinct local maxima, next to each other, are determined correctly. However, the robustness of the determination of droplet sizes below 2 µm is reduced, as the absolute scattering intensity of those small droplets is low. This leads to measurement data close to the lower limit of the dynamic range of the detection system, and the individual local maxima are less prominent. Moreover, as the distance between the maxima increases with decreasing droplet size, maxima within the evaluation region of the WALS-data (10–80°) might become cut off for droplets passing the measurement volume shifted from its center (see [23] for further details), leading to only one detectable peak. These data cannot be evaluated at present. The evaluation of the measured data with this method is exemplarily illustrated in Figure 2 for the superimposed scattering of an EtOH/TTIP-droplet and TiO_2_-nanoparticles.

**Figure 3:**
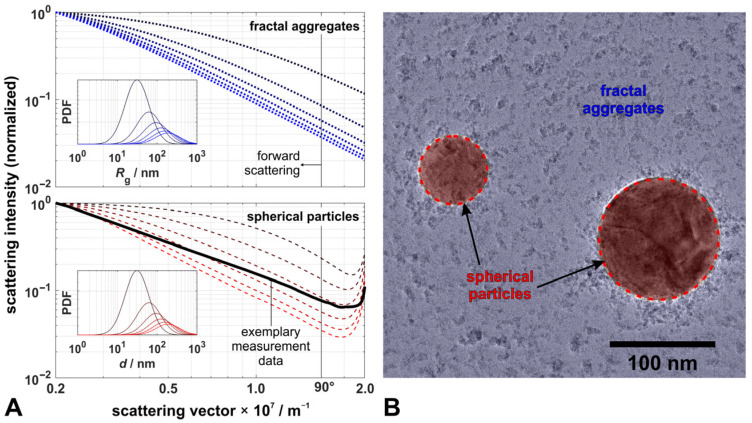
(**A**): Exemplary calculated scattering data for fractal aggregates (blue dotted lines) and spherical particles (red dashed lines) for log-normally distributed sizes (50 nm ≤ *μ*_g_ ≤ 300 nm, *σ*_g_ = 2.0) using RDG-FA (with *D*_f_ = 1.70) and Mie theory (with *n* = 2.540), respectively (data in double logarithmic scale, 90° scattering angle visualized). Geometric means *μ*_g_ refer to *R*_g_ for fractal aggregates and to *d* for spheres; the lower diagram includes exemplary experimental scattering data from the EtOH/TTIP-spray flame at 120 mm HAB (black line). (**B**): TEM image showing TiO_2_-particles sampled from the identical spray flame in spherical shape (red areas) and in fractal-like shape (distributed over blue area).

**Figure 4:**
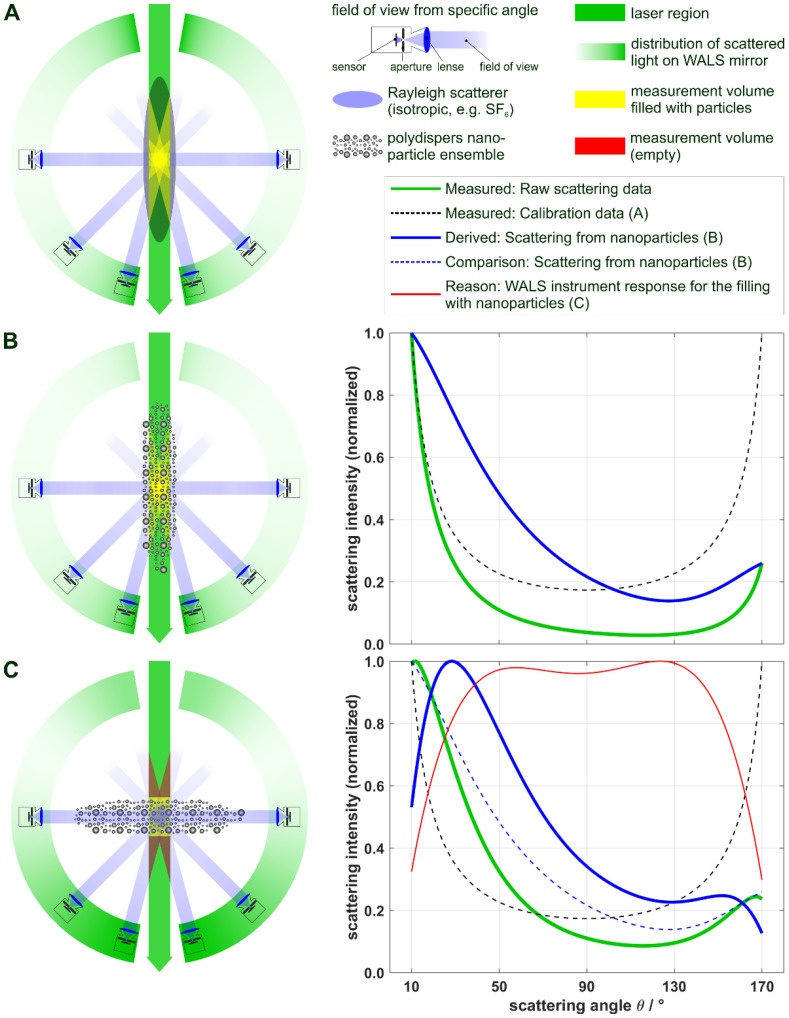
Illustration of WALS scattering patterns for isotropic scatterers (**A**) and a polydisperse particle ensemble (**B**), both filling the measurement volume homogeneously and completely; a non-homogeneous filling of the measurement volume and its characteristic scattering pattern is depicted in (**C**); scattering diagrams next to (**B**,**C**) show the raw scattering data (green), the calibration of the measurement volume from (**A**) (black dashed lines) and the derived scattering from particles (blue); in (**C**) the WALS instrument response (red) for this type of non-homogenous filling of particles is additionally given.

From the scattering image (Figure 2A) the scattering data are obtained for each angular section with the resolution of 0.2° (Figure 2B; for better illustration in logarithmic scale). Hereby, the radial extent of valid data within the scattering image was increased from 15 pixel to 40 pixel compared to our previous work, to increase the SNR [23]. In this example, 10 local maxima were determined within an angular range of 22.2°. Using the linear correlation between *d* and *ν* depicted in Figure 1B, a corresponding droplet size of 14.2 µm can be derived. In contrast to the calculated scattering data, the *peakfinder* function was conducted on normal measurement data, while the MPP was set to 0.06 (optimum value based on the results from a parameter study). The typically smooth and monotonously descending shape of nanoparticle scattering (e.g., Figure 3 and Figure 4 in Section 2.3) may introduce a slight shift in the positions of the maxima; however, their number within a certain angular interval is almost independent from any other underlying signal.

This evaluation method offers some advantages compared to the method described in our previous work. It can be conducted very quickly and is suitable for droplet sizes between 2 µm and 50 µm, as only the local maxima of the scattering data are relevant, which are mostly recognizable, even in the presence of scattering nanoparticles. Hence, the new method is, moreover, insensitive towards small angular shifts and signal cutoff caused by droplets passing the setup off-centered. This can be seen exemplarily in the scattering data presented in Figure 2. Here, the characteristic local maxima from a droplet only occur for scattering angles up to 40°, followed by a sudden decrease in intensity and a smooth shape of the scattering data up to 137°. The signal between 40° and 137° is significantly higher than the corresponding background level in this angular region and can be related to scattering from nanoparticles present in the measurement volume along with the droplet. For scattering angles between 10° and 40° and between 137° and 170°, the superposition of scattering from a single droplet and nanoparticle ensembles occurs. Thus, the new method allows for the determination of droplet size distributions, not only for pure solvent sprays, but also during nanoparticle synthesis in the spray flame. Additionally, this method is very robust towards uncertainty in the refractive index (e.g., caused by the mixture of solvent and precursor, heating and evaporation of the solvent in the flame), as an under- or overestimation in the refractive index of ± 10% results in deviations of less than 5.0% for droplet sizes > 2 µm (Figure 1B).

### 2.3. Evaluation of Scattering from Nanoparticles 

Depending on the operating conditions of the FSP-process and the solvent–precursor mixtures used, the formation of various particular systems is feasible, ranging from fractal-like aggregates to spherical particles [3,6]. For the gas-phase synthesis of TiO_2_-nanoparticle this statement is supported by several studies that have shown the formation of spherical and fractal particles in similar processes [28,29,30,31,32,33,34]; though the burner systems, operating conditions, and materials used, in some cases, differed to a certain degree from the ones in our work. Assuming particle sizes *D* of such processes as log-normally distributed [28,35] with the geometric mean *μ*_g_ and the geometric standard deviation *σ*_g_

(1) $$P\left(D,\mu_{g},\sigma_{g}\right)=\frac{1}{D\ln\sigma_{g}\sqrt{2\pi }}\cdot \exp\left[-\frac{{\left(\ln D-\ln\mu_{g}\right)}^{2}}{2\ln{\left(\sigma_{g}\right)}^{2}}\right],$$

the resulting scattering signal vector ***I***(*µ*_g_,*σ*_g_) of the size distribution can be computed by integration

(2) $$I\left(\mu_{g},\sigma_{g}\right)=\int_{D_{\min}}^{D_{\max}}P\left(D,\mu_{g},\sigma_{g}\right)\cdot I\left(D\right)\;dD,$$

where ***I***(*D*) is the calculated scattering intensity in the angular range of the WALS mirror. The superimposed scattering signal ***I***_C_ for both spherical and fractal particle fractions can then be calculated by

(3) $$I_{C}\left(\mu_{g,s},\sigma_{g,s},\mu_{g,f},\sigma_{g,f},\chi ,C\right)=\left\{\left[\chi \cdot I_{C,s}\left(\mu_{g,s},\sigma_{g,s}\right)+\left(1-\chi \right)\cdot I_{C,f}\left(\mu_{g,f},\sigma_{g,f}\right)\right]\cdot I_{\mathrm{CAL}}+I_{\mathrm{BG}}\right\}\cdot C$$

with the distribution parameters *µ*_g,s_ and *σ*_g,s_ for the fraction of spherical particles with diameter *d*, and *µ*_g,f_ and *σ*_g,f_ for the fraction of fractal aggregates with radius of gyration *R*_g_. Here, *χ* represents the ratio of the scattering contribution from spheres to the total signal and *C* is a scaling parameter. The scattering intensity from calibration measurements ***I***_CAL_ and background measurements ***I***_BG_ account for the scattering angle dependent size of the measurement volume and the background levels from camera, laser, and flame luminosity, respectively. Scattering data were calculated using a discretized form of Equation (2) and databases with discrete step sizes for each particle fraction (*d* ∈ [1 nm, 2000 nm], *R*_g_ ∈ [0.5 nm, 1000 nm]). Scattering for spherical particles was calculated using Mie theory following [27,36], assuming refractive indices of 1.000 for the surrounding gas atmosphere in the spray flame and 2.540 for TiO_2_-nanoparticles [37] and vertical polarized light at a wavelength of 532 nm. For fractal aggregates, scattering data were calculated using the confluent hypergeometric function _1_*F*_1_, based on the Rayleigh–Debye–Gans theory for fractal aggregates (RDG-FA) [15,22]. The fractal dimension was assumed constant with *D*_f_ = 1.7, as observed for fractals from the gas-phase synthesis of TiO_2_-nanoparticles in the literature [38]. Figure 3A illustrates the differences in the scattering diagrams in the range of scattering vectors accessible with WALS, between spherical (dashed red lines) and fractal TiO_2_-nanoparticles (dotted blue lines) for log-normally distributed particle sizes, with *μ*_g_ between 50 nm and 300 nm and *σ*_g_ = 2.0. Scattering from fractal aggregate ensembles monotonically decreases, whereas scattering from spheres exhibits an increase in intensity towards higher scattering vectors (and thus higher scattering angles). The latter pattern can be observed in WALS experiments on the EtOH/TTIP-flame (black solid line in Figure 3A), indicating the presence of spherical particles in the spray flame. Additionally to spherical particles, smaller fractal-like structures can be seen in the TEM images from samples taken in the EtOH/TTIP spray flame (see highlighted regions in Figure 3B), revealing that both spherical and fractal nanoparticles are actually formed in this process, and thus scattering from both fractions is superimposed.

Since the size of the measurement volume is also dependent on the scattering angle, the scattered light intensity from an ensemble of nanoparticles varies, not only with the angle and the size distribution, but also with the size of the measurement volume, following a 1/sin(*θ*) behavior, where *θ* is the scattering angle [20]. In the present work, this ranges from approximately 1 mm^3^ for 90° to 5 mm^3^ for 10° and 170° scattering angles, respectively. A reliable evaluation of scattering data from nanoparticles is thus only possible if they fill the measurement volume homogeneously (on average), like the gas molecules used for calibration (see above). Here, a highly turbulent system for particle synthesis is investigated, which does not only show high spatial and temporal fluctuations [39], but the superposition of scattering signals from spherical micro droplets and nanoparticles. Non-homogeneous filling of the measurement volume will then lead to false signal interpretations, as the conditions between the necessary calibration and the measurement are different. This is depicted in Figure 4, where the situation for calibration is schematically shown in A (blue elliptic area representing the region of the gas and yellow areas representing the filled measurement volume resulting from the intersection of the laser-beam and the region of observation under a certain angle; black dashed lines in diagrams B and C represent the calibration of A). Figure 4B shows the setup with a homogeneous particle filling of the measurement volume and the resulting raw scattering data (log-normally distributed TiO_2_-spheres with geometric mean *μ*_g_ = 100 nm and geometric standard deviation *σ*_g_ = 1.70), before calibration (green solid line) and after calibration (blue solid line). Figure 4C demonstrates the effect of non-homogeneous filling for a case of a thin aerosol sheet being cut perpendicularly by the laser. Here, the signal after calibration for small and large scattering angles is cut off (blue solid line), as the measurement volume is not completely filled with particles (red areas), leading to non-evaluable scattering data. As, in principle, an infinite number of non-homogeneous fillings exists, each one with a different ‘cut-off’ behavior (red solid line in Figure 4C representing the WALS instrument response for this specific type of particle-filling), it is a fundamentally ill-posed problem to simultaneously derive the measurement volume filling and the particles sizes from only the scattering signal.

Although this might be a rare and exaggerated example, a sound evaluation of particle size distributions with pulsed WALS, therefore, requires an appropriate filtering of the scattering data: first, only scattering data that can be attributed to particle scattering (avoiding interference with droplet scattering) must be filtered; second, an averaging of these filtered data is favorable, as the assumption of a homogeneously filled measurement volume (on average) is then fulfilled with a much higher probability. The strictly monotonic decrease of scattering intensity in the forward scattering direction (*θ* < 90°) is apparent for both RDG-FA and Mie theory (Figure 3) and is applied as filter criteria for scattering data from WALS single-shot measurements, to identify valid scattering from nanoparticle ensembles. For filtering, the monotony of calibrated scattering data in the range of 10° to 80° with a resolution of 3° is investigated. Data that do not exhibit a strict monotonic decline, e.g., due to non-homogeneous particle filling or superimposed scattering from droplets, are rejected. The filtering procedure is further refined by combining the monotony filter with a symmetry filter, taking into account the deviations in scattering behavior between the left and right mirror section. Here, valid scattering data must not exceed an average relative deviation, between both mirror sections, of 10%.

In order to obtain all quantities of interest (QoI), a weighted least-square method is applied. It is based on a Bayesian approach, comparable to studies on fractal aggregates [22], yet here including scattering from both particle fractions. For Gaussian distributed and independent noise (here referred to as uncertainty), the maximum likelihood estimate ***x***_MLE_ can then be computed by minimizing the squared weighted residual function

(4) $$x_{\mathrm{MLE}}=\arg\min\|{\left(\frac{I_{M}-I_{C}\left(x\right)}{\sigma_{I_{M}}}\right)}^{2}\|.$$

Here, ***I***_M_ is the measured scattering signal derived from the scattering image, ***I***_C_ is the calculated scattering signal using Equation (3) with ***x*** = (*µ*_g,s_, *σ*_g,s_, *µ*_g,f_, *σ*_g,f_, *χ*, *C*), and ***σ***_IM_ is the uncertainty of the measurement data. The latter is calculated from the standard deviation from the shot-to-shot variations. In this procedure, data are normalized by the integrated scattering intensity, to account for variations in absolute scattering intensity, due to fluctuations of laser pulse energy and total particle concentration, which we assume to be the predominant influence on variations in absolute shot-to-shot scattering intensity. As a result, interdependencies of the uncertainty occurring with these fluctuations are mostly cancelled out, and the remaining uncertainty can be considered as independent noise.

There are different numerical approaches for deriving ***x***_MLE_ or the posterior probability *p*(***x***|***I***) representing the probability density for parameters ***x*** by given measured data ***I*** [40]. A straight forward calculation of *p*(***x***|***I***) for various ***x*** is theoretically possible but not feasible in our work, as the computation time increases with both increasing parameters in ***x*** and upper and lower boundaries and variable increments, respectively. Therefore, we use two different numerical approaches, as in Huber et al. [22]. The first method is a non-linear gradient-based solver (MATLAB function *lsqnonlin*) that derives ***x***_MLE_ starting from an initial parameter set ***x***_0_ by least-square minimization (subsequently referred to as the least-square method). As, in some cases, the derivation of ***x***_MLE_ shows a dependency on the initial parameter set, we here extend the method by a parent optimization structure: to obtain ***x***_MLE_ we start from multiple initial points ***x***_0,*j*_ (*j*: number of starting points, here *j* = 50) randomly distributed within the defined boundary conditions (see below). This method includes the derivation of the Jacobian matrix, from which the credibility interval of ***x***_MLE_ can then be derived [22]. As a second method, a Markov Chain Monte Carlo algorithm (subsequently referred to as the MCMC method) is used. Here, starting from ***x***_0_ a random next vector ***x***_1_ is generated, which is accepted or rejected according to its probability (Metropolis–Hastings criterion). Iteratively, a series *N*(***x***_0_, ***x***_1_, …, ***x****_n_*) is generated, which becomes ergodic in *p*(***x***|***I***) for a sufficiently large *n* and from which point estimates for ***x*** can be derived [22]. We use an algorithm developed by Grinsted et al., based on the Goreman–Weare-method, which is optimized to achieve good convergence even for badly scaled or high-dimensional problems [41,42,43]. As this algorithm allows several starting points (so-called walkers) and parallel computing of each iteration, a comparably fast computation of results is possible, even for large *n*. In this work 50 walkers, each with a length of 20.000 individual steps, were used, resulting in an overall sample size of 10^6^ for each QoI.

Lower and upper boundaries ***x***_L_ and ***x***_U_ can be artificially included in the weighted residual function using Heaviside step functions *Θ*(***x*** − ***x***_L_) and *Θ*(***x***_U_ − ***x***), respectively. The boundaries were set to 20 nm ≤ *µ*_g,s_ ≤ 500 nm and 10 nm ≤ *µ*_g,f_ ≤ 250 nm for the geometric mean of spheres and fractals, respectively, to 0 ≤ *χ* ≤ 1 for the scattering ratio, and to 0 ≤ *C* ≤ 2 for the scaling parameter. As the databases used for calculation of scattering signals from both spherical and fractal particle fraction are limited in lower and upper particle sizes, the possible combinations of distribution parameters (*µ*_g_,*σ*_g_) obtaining a full size distribution are limited (i.e., combinations towards larger *µ*_g_ and *σ*_g_ lead to a cutoff at the upper end of the distribution). To avoid too large cutoffs, all possible combinations (*µ*_g_,*σ*_g_) with less than 99% coverage of the respective full-size distribution were discarded. To that end, we included the inequation *σ*_g_ < *a*∙(*µ*_g_/µm)^−0.43^ (with *a* = 26.2 for the database of spheres and *a* = 19.5 for fractals) representing a functional relation between the upper bounds for the geometric standard deviation and the geometric mean.

## 3. Results and Discussion

In the following sections, we first compare results from droplet sizing in a pure EtOH spray flame without nanoparticle formation, utilizing the method based on the residuals of our previous work [23] and the one based on the local maxima described in Section 2.2. We then demonstrate the applicability of this method for superimposed scattering data from droplets and nanoparticles in an EtOH/TTIP spray flame and present the first results from nanoparticle sizing for both spheres and fractals.

### 3.1. Droplet Size Distributions

**Figure 5:**
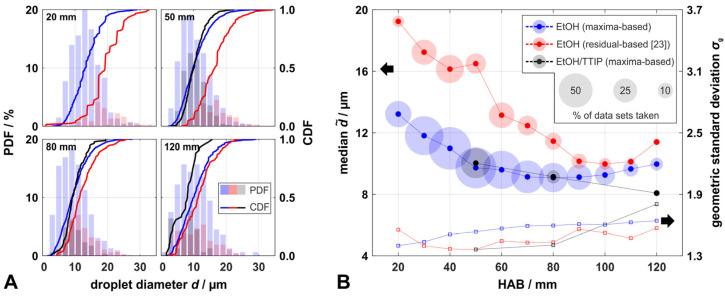
Comparison of results from droplet sizing at an EtOH spray flame using the novel maxima-based method (blue colored plots) and the residual-based method of our previous work [23] (red colored plots); results from a particle-laden EtOH/TTIP spray flame are additionally shown in grey/black. (**A**): relative number frequency distributions (PDF) and their cumulative distributions (CDF) of droplets measured at the center axis of the SpraySyn flame for different HAB; (**B**): median $\tilde{d}$ and geometric standard deviation *σ*_g_ derived from droplet distributions, the diameter of the circle corresponds to the percentage of evaluable data sets at a specific HAB.

Figure 5A shows exemplary droplet size distributions at four different HAB from the EtOH spray flame, derived from the novel maxima-based method (blue) in comparison to those derived from the residual-based method of our previous work [23] (red). To demonstrate the applicability of the novel method for superimposed data from droplets and nanoparticles, we conducted measurements at an EtOH/TTIP spray flame at selected HAB (grey/black). Relative size distributions at each HAB were normalized by the amount of droplets derived from the maxima-based method in the EtOH spray flame, to visualize deviations between the different methods and flame types. Figure 5B shows the median values $\tilde{d}$ and geometric standard deviations *σ*_g_ derived from droplet distributions for a HAB between 20 mm and 120 mm. The circle size in Figure 5B indicates the relative amount of data sets evaluable at a specific HAB. 

Results from the computer-based evaluation procedure for both methods were manually checked for erroneous data sets, to avoid an over- or underestimation of the derived droplet sizes; data sets with erroneous detection of local maxima for the maxima-based method and data sets with an erroneous data fit for the residual-based method were discarded. The novel method was significantly less prone to errors in the computer-based evaluation, as only 16% of all data sets taken from the EtOH spray flame for all HAB were discarded, compared to 51% for the residual-based method. Thus, especially towards smaller droplet sizes for all observed HAB, the maxima-based method was more likely to detect droplets than the residual-based method. This led to a broadening of the distributions, to a range of *σ*_g_ between 1.40 and 1.64, and a decrease in $\tilde{d}$, to a size range between 9.1 µm and 13.2 µm. The larger sample sizes (e.g., 1775 droplets from the maxima-based method compared to 382 droplets from the residual-based method at 50 mm HAB) had a notable positive effect on the derived statistics. Both $\tilde{d}$ and *σ*_g_ derived from the maxima-based method showed a smoother profile with increasing HAB than the ones derived from the residual-based method. Between 20 mm and 80 mm HAB, $\tilde{d}$ decreased from 13.2 µm to 9.1 µm and then slightly increased to 10.0 µm up to 120 mm. Following the results from the PDA measurements in a similar SpraySyn-flame conducted by Stodt et al., increased evaporation speeds for smaller droplet sizes (*d*^2^-law) led to an increase in the mean droplet size, from 20 mm to 80 mm HAB in their flame, which agrees with our observations, although at larger HAB >80 mm [24]. For lower HAB our data show contradictory behavior compared to the results of Stodt et al., indicating the decreased sensitivity of our methods towards smaller droplets in lower regions of the spray flame with higher spray density. Independent of the evaluation method, scattering from small droplets could hardly be distinguished from the superimposed scattering from larger ones. In such cases, only the size of the largest droplet was determined.

Droplet-measurements at the EtOH/TTIP-flame were conducted at 50 mm, 80 mm, and 120 mm HAB, where a significant contribution of nanoparticles was observed in the scattering data, and thus superposition of both fractions occurred. The results show a small increase in $\tilde{d}$ (up to +3.1%), but narrower size distributions (Δ*σ*_g_ down to −12.0%) compared to the corresponding reference values of the pure EtOH spray at 50 mm and 80 mm HAB. In a particle-laden spray flame, the sensitivity towards smaller droplets is presumably decreased by the superposition of droplet and particle scattering, lowering the prominence of the local maxima of the droplets; similar to a lower HAB, at which superposition of several droplets occurs. In such conditions, scattering from small droplets and their relatively low peak prominence are hardly distinguishable from the overall scattering intensity. This leads to a narrower size distribution and to a significantly lower number of detected droplets than for pure droplet scattering, which requires a relatively long measurement time to generate a sufficiently large sample size. With the presence of nanoparticles, 47% to 76% less images were evaluable compared to the results from the pure EtOH spray flame. However, at 120 mm HAB, the opposite tendencies occurred: despite the fact that a greatly reduced sample size was captured, the size distribution broadened towards smaller droplet sizes ($\tilde{d}$ = 8.1 µm, *σ*_g_ = 1.81) and showed bimodality (Figure 5A). This may have been caused by micro-explosions of the evaporating and burning EtOH/TTIP-droplets, as observed by Li et al. in their investigations of single droplet combustions using a similar type of mixture, yet with a higher molar concentration of TTIP [34]. According to their studies, the significantly higher evaporation rate of EtOH leads to an increase in the concentration of TTIP, mainly towards the surface of the droplet, which causes local superheating of EtOH and results in the explosion of the droplet. Assuming an evaporation of approx. 60% of the EtOH component before micro-explosions occur [34], the average refractive index of a droplet changes by less than 1% [44], resulting in a negligible impact on the derivation of droplet sizes with our novel method.

### 3.2. TiO_2_ Particle Size Distributions

**Figure 6:**
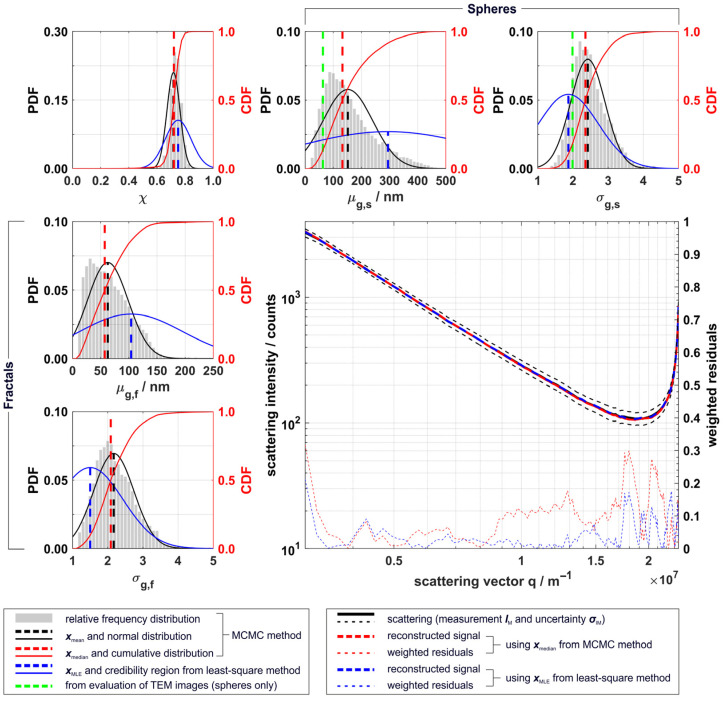
Top row and left column: Results for distribution parameters of spheres, fractals, and scattering intensity ratio from MCMC and least-square methods for particle scattering from an EtOH/TTIP spray flame at 120 mm HAB. Each of these diagrams shows the relative probability distributions (PDF) from the MCMC method (grey bars), its arithmetic mean ***x***_mean_ and normal distribution fit (black), its median ***x***_median_ and relative cumulative distribution (CDF, red), and the ***x***_MLE_ within its credibility region, derived from least-square method (blue); results from TEM image analysis for spherical particles are shown in green. Bottom, right: measured data ***I***_M_ (black) within the uncertainty of the measurement ***σ***_IM_ (black dashed lines) and the reconstructed scattering data using Equation (3) and ***x***_median_ of the MCMC distribution (red) and ***x***_MLE_ of the least-square method (blue); ***σ***_IM_-weighted residuals for both reconstructions are shown in respective colors on the right *y*-axis.

Results for particle size distributions derived with the MCMC method and the least-square method from filtered and averaged scattering data measured at HAB 120 mm were obtained following Equation (3) and are shown in Figure 6. For each QoI the relative probability density distribution (PDF), its normal distribution fit, the cumulative probability distribution (CDF), and the derived point estimators (namely the arithmetic mean ***x***_mean_, the median ***x***_median_ from the MCMC-method, and the ***x***_MLE_ within its credibility region derived from the Jacobian matrix from the least-square method) are shown; the larger diagram contains the measured scattering data and reconstructed data based on ***x***_median_ and ***x***_MLE_ and their weighted residuals. Particle size distribution parameters derived from TEM image analysis could only be obtained for spherical particles, as fractal particles were, on the one hand, not sufficiently distinguishable from the TEM background and, on the other hand, not clearly separated from each other on the TEM grid (compare Figure 3).

The distributions obtained from the MCMC method were (in good approximation) normally distributed for most QoI, obtaining nearly similar values for ***x***_median_ and ***x***_mean_ (see also Table 1). Only for *µ*_g,s_ and *µ*_g,f_ did the results show a positively skewed distribution, resulting in a smaller value for the distribution median (132.6 nm for *µ*_g,s_ and 57.2 nm for *µ*_g,f_) compared to the arithmetic mean (151.9 nm for *µ*_g,s_ and 62.6 nm for *µ*_g,f_). The probability distributions obtained from both methods were broad, except for *χ*, revealing the ill-posedness of a multi-variate data analysis of the given problem; especially arising from the two morphological particle fractions being present and the relatively high uncertainty of the scattering data arising from the turbulent character of the process. The ill-posedness could be greatly reduced for synthesis conditions with only one morphological fraction present. ***x***_MLE_ differs from ***x***_median_ for nearly all QoI, although within *p*(***x***|***I***) derived from the MCMC. However, the reconstruction of the scattering data based on the two methods was in both cases very accurate. A more detailed analysis of single results within the parent optimization structure of the least-square method revealed that for 20% of the randomly distributed starting points different ***x***_MLE_ were obtained, indicating the presence of local minima in the residual function for specific parameter combinations ***x***. The least-square method derived a point estimate ***x***_MLE_ by minimizing the squared weighted residual function of Equation (4), employing a gradient-based solver. It is, thus, sensitive towards such local minima, and differing ***x***_MLE_ may be obtained when different starting points are used. However, the ***x***_MLE_ obtained by the other 80% of the starting points led to the best reconstruction of the data within the uncertainty of the measurement (residuals smaller than 0.2∙***σ***_IM_).

The MCMC method is generally more trustworthy, as it generates a chain representing the probability distribution *p*(***x***|***I***), from which a representative ***x*** like ***x***_median_ can be derived, and it is comparably robust, even with the presence of local minima in the residuals. Moreover, credibility intervals, and thus uncertainty estimates, can be directly determined for each of the parameters from the distribution. Though these results differed from ***x***_MLE_, the reconstruction of scattering data was also very accurate and within the uncertainty of the measurement (residuals smaller than 0.3∙***σ***_IM_). For further discussion of particle size distributions obtained at different HAB, we therefore compare the QoI based on ***x***_median_ and ***x***_mean_, including their standard deviation derived from the MCMC method. The results for all relevant QoI (excluding the scaling parameter *C*) at 50 mm, 80 mm, and 120 mm HAB are listed in Table 1, including *µ*_g_ and *σ*_g_ from log-normal fit based on TEM image analysis for spherical particles at 120 mm HAB.

Between 50 mm and 120 mm HAB, the spheres increased in *d* by approx. 14%, while the *R*_g_ of the fractals decreased by ~56%. This indicates both the growth and coalescence of spherical particles and the sintering of fractal structures to spherical particles in the hot environment of the spray flame, similarly to what Huber et al. observed for fractal silica aggregates at high temperatures in a furnace tube [21]. A further indication for this assumption is provided by the light scattering ratio, which yielded a significantly higher fraction of spheres for a HAB > 80 mm. The widths of the size distributions for both particle fractions showed no significant deviations with increasing HAB, whereby the distributions for spheres were generally broader (between 2.36 to 2.58) than the ones for fractals (between 1.84 and 2.19). The log-normal fit parameters derived from TEM data for spheres were significantly lower than the respective values derived from the MCMC, especially for the mean particle size (−52% for *µ*_g_, −16% for *σ*_g_), yet were still within one standard deviation of the derived normal probability distribution *p*(***x***|***I***) (Table 1 and Figure 6, top row, middle, and right diagram). 

However, the integrated angular distribution of elastically scattered light from a distribution of nanoparticles is very sensitive towards large particles. Results from both scattering experiments and TEM showed relatively broad distributions, with *σ*_g_ between 2.0 and 2.6, indicating that in a turbulent process such as FSP, comparably large particles are also generated and measured. Regarding the discrepancy of both results, there are two possible explanations: First, the TEM samples were collected with a total integration time of 10 s, the summed WALS-exposure time after averaging was in the order of a few µs (6 ns laser pulse width). As the scattering intensity of large particles is much stronger compared to small ones, a few large particles randomly present in the WALS-measurement volume during a laser pulse might have altered the results. This effect is possible, especially under such turbulent conditions. Second, as we could show above, at large HAB, a small amount of droplets was still present that probably underwent micro-explosions, leading to the bimodal size distribution. Secondary droplets of such explosions with diameters below 1 µm could be superimposed with the particle scattering, without being noticed, as those droplets would not show prominent maxima. Those data could pass the data filter and contribute to the average WALS-scattering data, leading to biased results for the spherical particle fractions (larger median and geometric width).

As both spheres and fractal aggregates with very small primary particle sizes are visible in the TEM images, particle formation for both the gas-to-particle and droplet-to-particle routes occurs [1]. According to Jossen et al., the formation pathway is mainly dependent on two criteria: (1) the combustion enthalpy density (CED) of the solvent-precursor solution in the gaseous atmosphere of the spray flame, and (2) the ratio of the boiling temperature of the solvent *T*_bp,s_ to the melting or decomposition temperature of the precursor *T*_d/m,p_ [45]. High CED > 4.7 kJ/g_Gas_ enhances the production of fine and homogeneously distributed nanoparticles, for smaller CED the temperature ratio is decisive, leading to gas-to-particle conversion for ratios *T*_bp,s_/*T*_d/m,p_ > 1.05. Although TiO_2_-particles were not produced in these experiments in the literature, the criteria may also apply to them, as comparable metal oxides such as SiO_2_, CeO_2_, and Al_2_O_3_, with similar formation pathways, were investigated [45]. For the EtOH/TTIP spray flame under investigation in this work a CED of 3.5 kJ/g_Gas_ and a temperature ratio of 1.21 (based on the boiling point of EtOH *T*_bp,EtOH_ = 351 K and the melting temperature of TTIP *T*_m,TTIP_ = 289 K) were obtained [3]. Following the results of Jossen et al., fractal aggregates consisting of homogeneous particles are more likely to occur [45]. However, Akurati et al. mentioned that the temperature ratio of Jossen et al. was significantly smaller when taking the boiling temperature into account instead of the melting temperature [29]. With *T*_bp,TTIP_ = 511 K (closer to its decomposition temperature of around *T*_d,TTIP_ = 523 K [46]) this results in a reduced temperature ratio of 0.69 for the mixture of EtOH and TTIP. At this lower temperature ratio, the formation of non-homogeneously distributed (spherical) particles is preferred, leading to comparably broad size distributions, between 2.36 to 2.58, in our research. The morphology of TiO_2_-nanoparticles from spray flame processes also shows a strong dependency on the synthesis process, as can be seen in the literature. Chang et al. used an identical solvent–precursor system in a diffusion flame burner for synthesis and observed that the homogeneity of particle sizes was also dependent on the molar concentration of the precursor [47]. At a concentration of 0.1 M TTIP (as used in this work), they observed the formation of fractal aggregates with very fine primary particles (~12 nm diameter on average). Spherical particles comparable to our work, however, were only obtained at concentrations above 0.5 M [47]. Measurements using another type of FSP-burner, with an acetylene/oxygen pilot flame, were carried out by Akurati et al. for WO_3_/TiO_2_ composite-particles. They obtained similar results to Chang et al., yet, for an even higher TTIP concentration of 2.0 M [29]. Li et al. provided a more suitable explanation for the formation of both spherical and fractal TiO_2_-nanoparticles from EtOH/TTIP droplets, based on their single droplet combustion experiments [34]. They assumed that during the evaporation of the mixture the concentration of TTIP on the outer shell of the droplet is increasing, as EtOH evaporates much faster than TTIP and the mass diffusion of EtOH from the droplets’ core to their surface is much slower than the evaporation rate. This leads to the formation of a layer around the droplets with high TTIP concentrations, from which (1) large particles could be formed in a hydrolysis reaction, with water vapor stemming from the combustion of evaporated EtOH, and (2) fractal aggregates could form from vaporized TTIP via the gas–phase route [34].

However, a direct comparison of our results with the literature is difficult, as most works differed in their synthesis processes, materials, or process conditions. Regardless, the presence of both morphological fractions measured by WALS in this work is clearly confirmed by our own TEM-analysis of the process, suggesting that WALS-results can be considered reliable, even under such complex process conditions.

## 4. Conclusions

In the present study, wide-angle light scattering (WALS), utilizing a pulsed laser source, was used for the determination of both droplet and particle size distributions from a standardized spray flame process (SpraySyn burner). Spray flames fed by pure ethanol (EtOH) and a solution of 0.1 M titanium isopropoxide (TTIP) in EtOH to incept the formation of titanium dioxide (TiO_2_) nanoparticles were investigated. 

The development of an improved evaluation routine, for the determination of droplet sizes up to 50 µm in spray flame synthesis, was successfully accomplished, and the novel method is applicable, even for superimposed scattering from droplets and nanoparticles. It is based on the number of scattering maxima and allows a very robust evaluation of a significantly larger number of scattering data sets compared to the residual-based method, leading to increased sample sizes and better statistical interpretation of the obtained droplet size distributions. Besides this, it is also very robust against small angular shifts in the data, which may be caused by (1) droplets measured off-center of the WALS measurement volume, and (2) uncertainties in the refractive index caused by, e.g., partial de-mixing of solvent mixtures, induced by evaporation. 

For a reliable evaluation of particle scattering data, sophisticated filtering and averaging of single-shot data was applied. As TEM images and WALS scattering patterns revealed the presence of both spherical and fractal-like TiO_2_-nanoparticles, both fractions had to be included in the evaluation of scattering data. Such high-dimensional multivariate analysis requires statistical evaluation methods. A Markov Chain Monte Carlo simulation obtained significantly more stable and reliable results than gradient-based non-linear regression. Despite the comparably high uncertainties of the results, which are typical for such multi-variate systems, we want to point out that the WALS technique (1) is capable of measuring size distributions of both microdroplets and nanoparticles and (2) can identify the presence of morphological fractions such as spheres and fractal aggregates simply from the shape of the scattering data. 

The combination of both methods offers essential insights into the evaporation behavior of the solvent and precursor and the particle formation pathways and, thus, a more comprehensive investigation of spray flame synthesis. A systematic examination at different heights above burner surface (HAB) obtained generally larger droplets towards greater HAB in the pure EtOH spray flame, since smaller droplets evaporate much faster. For droplets containing TTIP, on average, smaller droplet sizes were measured, presumably caused by micro-explosions induced by the precursor in the mixture. The formation of both spherical and fractal particles was mainly incepted by the different evaporation and diffusion behaviors of EtOH and TTIP. With increasing HAB, we could observe the growth of the spherical particles by coalescence and, most likely, sintering of fractal aggregates.

In future research, a detailed investigation of the TTIP precursor system employed with the SpraySyn burner, using different solvents and molar concentrations of the precursor, will further contribute to a better understanding of the underlying evaporation and particle formation mechanisms. Moreover, the technique will be applied to other solvent–particle systems, such as EtOH/2-Ethylhexanoic acid and Iron(III)-nitrate, leading to nanoparticles with very high specific surfaces.

## Figures and Tables

**Table 1:** Distribution Parameters for Spherical and Fractal Nanoparticle Fractions and their Scattering Ratio Derived from Scattering Experiments at 50 mm, 80 mm, and 120 mm HAB, Utilizing the MCMC Method (values in normal font: QoI based on ***x***_median_; values in italics: QoI based on ***x***_mean_ and their standard deviation); Data from TEM Analysis at 120 mm HAB Represent *µ*_g_ and *σ*_g_ from a Log-Normal Fit (spheres only).

HAB/mm	Spheres		Fractals		*χ*
	*μ*_g,s_/nm	*σ*_g,s_	*μ*_g,f_/nm	*σ*_g,f_	
50	95.8 (*124.3* ± *98.0*)	2.36 (*2.42* ± *0.50*)	131.0 (*121.0* ± *58.3*)	1.84 (*2.11* ± *0.89*)	0.55 (*0.56* ± *0.12*)
80	103.4 (*130.1* ± *95.5*)	2.58 (*2.66* ± *0.59*)	60.9 (*67.4* ± *43.1*)	2.19 (*2.36* ± *0.85*)	0.75 (*0.74* ± *0.07*)
120	132.6 (*151.9* ± *88.0*)	2.36 (*2.42* ± *0.49*)	57.2 (*62.6* ± *36.3*)	2.09 (*2.17* ± *0.57*)	0.72 (*0.72* ± *0.05*)
120 (TEM)	63.3	1.99	-	-	-

## Data Availability

The data presented in this study are available on request.
